# Effectiveness and cost-effectiveness of community-based mental health services for individuals with severe mental illness in Iran: a systematic review and meta-analysis

**DOI:** 10.1186/s12888-024-05666-7

**Published:** 2024-04-04

**Authors:** Mozhgan Taban, Sara Nooraeen, Kiarash Tanha, Maziar Moradi-Lakeh, Seyed Kazem Malakouti

**Affiliations:** 1 Mental Health Research Center, Psychosocial Health Research Institute, Iran University of Medical Sciences, Tehran, Iran; 2 Department of Psychiatry and Psychology, Mayo Clinic, Rochester, MN USA; 3 Oxford Vaccine Group, Department of Pediatrics, University of Oxford, Oxford, U.K.; 4 Gastrointestinal and Liver Disease Research Center (GILDRC), Iran University of Medical Sciences, Tehran, Iran; 5 Geriatric Mental Health Research Center, School of Behavioral Sciences and Mental Health, Iran University of Medical Sciences, Tehran, Iran

**Keywords:** Effectiveness, Cost-effectiveness, Community-based mental health services, Severe mental illness

## Abstract

**Background:**

Severe mental illness (SMI) imposes a substantial worldwide burden of disability, highlighting the need for comprehensive and adaptable mental health services. This study aims to assess the efficacy and cost-effectiveness of community-based mental health services (CBMHS) in reducing relapse and rehospitalization rates among individuals with SMI in Iran.

**Method:**

A systematic review and meta-analysis were conducted. Medline, EMBASE, ISI, SCOPUS, and ProQuest were searched until December 2022. We focused on randomized controlled trials, quasi-experimental studies, or economic studies related to individuals with SMI. Out of 127 articles, 17 were selected for a full-text review. The primary outcomes were the severity of psychopathology, rehospitalization rates, and the mental health of caregivers. We also examined community-based interventions and their impact on various outcomes. Data extraction and risk of bias assessment were performed, and critical appraisal was conducted using JBI checklists. Meta-analysis was carried out using STATA software. (PROSPERO registration. CRD42022332660).

**Result:**

Rehospitalization rates among patients who received CBMHS were significantly lower, with an odds ratio of 2.14 (95% CI: 1.44 to 3.19), indicating a 2.14 times lower likelihood than those who received treatment as usual. A reduction in psychopathology accompanied this, SMD: -0.31, 95% CI: -0.49 to -0.13, I2 = 40.23%). Moreover, there was a notable improvement in social skills (SMD: -0.7, 95% CI: -0.98 to -0.44, I2 = 0.00%). The burden on caregivers also decreased (SMD: -0.55, 95% CI: -0.99 to -0.1, I2 = 63.2). The Incremental Cost-Effectiveness Ratio (ICER) for QUALY was acceptable, albeit with a wide range of 613 to 8400 Dollars.

**Conclusion:**

CBMHS has demonstrated effectiveness and efficiency in Iran as a developing country. Additionally, it shows promise in mitigating the shortage of acute psychiatry beds. Using multiple data collection tools poses a limitation regarding data consolidation and conducting a meta-analysis.

**Supplementary Information:**

The online version contains supplementary material available at 10.1186/s12888-024-05666-7.

## Introduction

Severe mental illness (SMIs) is a prevalent global cause of disability. In 2019, mental disorders contributed to approximately 418 million disability-adjusted life years (DALYS), accounting for 16% of global DALYS. This represents a significant increase compared to previous estimates. The World Health Organization's (WHO) Action Plan (2013–2030) emphasizes establishing inclusive and adaptable community-based mental health and social care services. The goal is to empower individuals affected by these disorders to exercise their full human rights and gain timely access to culturally appropriate, high-quality healthcare and social support. This approach promotes recovery, enables individuals to achieve optimal well-being, actively participate in society and employment, and eliminates stigmatization and discrimination [1].

Although medication is essential for symptom control and relapse prevention, it is insufficient to address the social needs of patients with severe mental illness. In recent years, there has been notable progress in pharmacotherapy, particularly in managing the acute phase of the disorder, which has subsequently increased the inclination toward providing community-based mental health services (CBMHS) [2, 3]. The various aspects of psychiatric disorders, including their health, familial, social, and economic dimensions, also highlight the different CBMHS, such as home visits, outpatient services, community-based rehabilitation, psychological training, family therapy, and other methods. Additionally, it acknowledges the financial burden that SMIs place on families and governments, with an estimated economic impact of approximately USD 5 trillion in 2019 [4].

Based on the findings of the Iranian Mental Health Survey (IranMHS) conducted in 2011, nearly a quarter of the population experiences psychiatric disorders, of which 3 to 5 presents suffer severe illness [5]. Furthermore, it emphasizes that these conditions are the leading cause of disability among individuals aged 10 to 40 in Iran [6].

Studies demonstrate that the provision of CBMHS effectively reduces relapse and rehospitalization rates in patients and alleviates the burden on families. Additionally, by lowering hospitalization and daycare costs, the economic burden associated with SMIS is reduced. However, offering these services in Low- and Middle-Income Countries (LMICS) encounters obstacles related to social, cultural, and financial factors [7]. In Iran, notable attention has been given to providing comprehensive, integrated, and responsive mental health services in community settings [8]. Conducted in Iran, it demonstrated a 67% reduction in hospitalization rates after individuals received CBMHS [9]. There are numerous studies conducted worldwide that have demonstrated the effectiveness and cost–benefit of community-based services [10–13]. However, in parallel with the expansion of community-based services, long-term hospitalization and asylum-like services were expanded nationwide in the last two decades. Our intention in this study was not to evaluate these services at the global level, but we checked whether this system works in Iran's cultural, social, and economic conditions, and maybe this Rio systematic is useful for mental health policymakers and shows that the development of these services requires It takes more effort. For this reason, we focused on Iranian studies.

To address the knowledge gap, we conducted a systematic review and meta-analysis of Randomized Clinical Trials (RCTs), quasi-experimental studies, or economic studies to evaluate the cost-effectiveness and cost benefit effectiveness of CBMHS in reducing relapse and rehospitalization rates among patients with SMIs. The findings of this study may have implications not only for future research in Iran but also for neighboring countries.

## Methods

### Eligibility criteria

RCTs, quasi-experimental studies, or economic studies conducted on specific outcomes such as clinical relapse, rehospitalization, cost, and the severity of psychopathology, were included. The intervention on individuals with a diagnosis of psychotic spectrum disorder (schizophrenia, schizophreniform, and other long-lasting psychotic disorders), Bipolar Mood Disorders (BMD), or severe refractory major depression were considered were considerd.

Amongst them, studies with any community-based intervention, including home-visit services (by professionals or peers and family members), telephone follow-up, family psychoeducation, and skill training, and the studies which aim the caregivers' knowledge, burden, and mental conditions as by proxy groups to have an impact on SMI clinical outcome were included.

Rehospitalization, relapses, clinical condition and severity of the symptoms, treatment adherence, economic outcomes including QALYS, CER (Cost-Effectiveness Ratio), social functioning, quality of life, and family knowledge were outcomes of interest.

Exclusion criteria were any studies performed on individuals with substance use disorders, intellectual disability, brain trauma, or; the intervention model was not transparent and did not have a follow-up interval.

The study protocol had been approved by the Ethics Committee of the Iran University of Medical Sciences (code: IR.IUMS.REC.1400.733) and registered in PROSPERO. (CRD42022332660, available here: https://www.crd.york.ac.uk/prospero/display_record.php?ID=CRD42022332660.)

### Search strategy

We systematically reviewed the published literatures on the intervention models for SMIs in Iran. This review includes all RCTs, quasi-experimental studies, or economic studies reporting on the effectiveness and cost-effectiveness of community-based care and interventions designed to promote social engagement among individuals with SMI.

The search was conducted for articles published in English using databases such as Medline, EMBASE, ISI, SCOPUS, and ProQuest. Additionally, peer-reviewed papers in the Persian language, were accessed via Iranian websites including SID, MAGIRAN, and Iran doc. Our search terms (keywords and Mesh terms) reflected central concepts: severe mental illness, models of intervention, outcome, and relapse. We limited our search to publications in English and Persian available in full text. If the full text was not available, the authors were contacted. publications included in the study were published until December 2022, the complete search strategy can be found in the Supplementary Material 1: Appendix.

Since this study aimed to estimate the effectiveness of CBMHS, and some of the included articles used alternative versions of the questionnaires, we excluded the data from alternative versions. We extracted the relevant data by carefully studying the tables and text.

### Screening and data extraction

Two independent reviewers (S.N., M.T.) assessed article titles and abstracts to exclude unrelated records. The full text of the remaining studies was also reviewed independently by S.N. and M.T., with unrelated articles being excluded. Any disagreements were resolved through discussion and judgment by the principal investigator (SK.M.).

A pre-designed data sheet was completed for each of the included studies. Data extraction from each included paper was performed by two independent authors (S.N., M.T.) based on the author's name, publication year, journal name, study population, city of the study population, sex, sample size, type of intervention, and tools.

### Risk of bias assessment

The relevant JBI critical appraisal checklist regarding the study designs (i.e., RCT, quasi-experimental, and economic evaluation) was used to evaluate the articles. JBI critical appraisal Checklist for RCTs has 13 questions evaluating different methodological aspects of an RCT, including randomization, concealed allocation, blinding, follow-up, and analysis (all versions of JBI available: https://jbi.global/critical-appraisal-tools).

### Statistical analysis

To ensure comparable results, we calculated Standardized Mean Differences (SMD) and 95% confidence interval between the intervention and control groups [10]. SMDs were calculated, where available, to assess the intervention's effectiveness during the follow-up period (i.e., a pre-post comparison in the experimental group) and to measure the differences between the experimental and control groups at the follow-up time (i.e., calculated as the post–pre-experimental mean minus the post–pre control mean). The odds ratio and 95% confidence interval were calculated to compare the rehospitalization rate between the groups.

The data were analyzed using STATA, version 17.0 (STATA Corporation, College Station, TX, USA). The statistical heterogeneity between the studies was assessed using the I2 statistic, which was able to measure the inconsistency across the results of the studies and describe the proportion of the total variations based on their estimates due to the presence of heterogeneity rather than sampling errors. A random-effects model was used if heterogeneity was observed (the I2 values > 50).

## Result

**Fig. 1:**
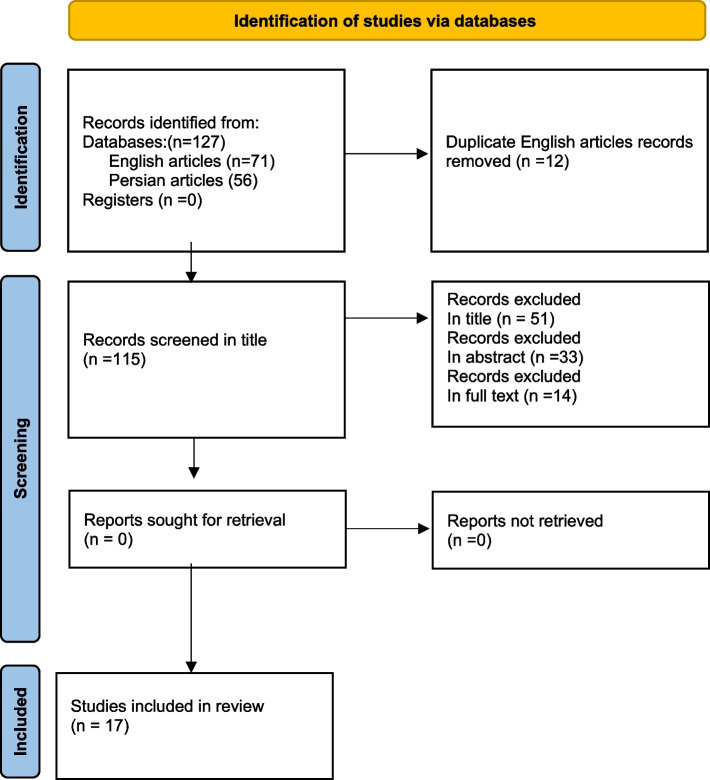
PRISMA flow chart to illustrate the article search and selection process

Seventy-one articles were selected through the English-language website, and fifty-six Persian-language articles were selected through the Iranian website. After removing duplicates, 115 articles remained. In the next steps, the titles and abstracts were reviewed, and 84 articles were excluded. The full text of thirty-one articles was reviewed, of which 14 unrelated articles were excluded. Finally, 17 English and Persian articles were included in the study (Fig. 1).

### Critical appraisal and risk of bias assessment

**Fig. 2:**
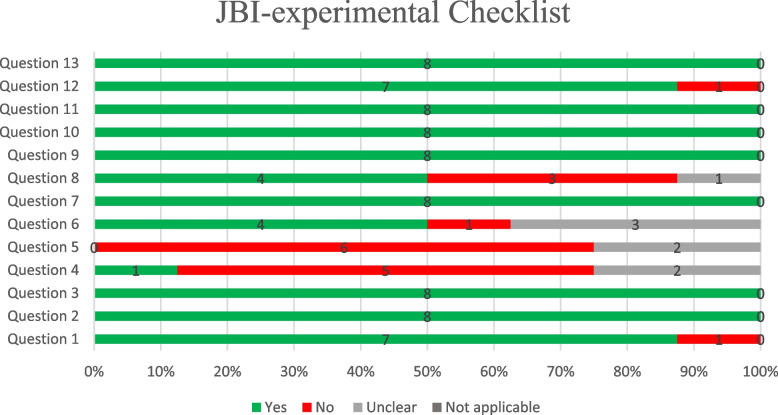
Quality assessment of experimental studies

The results of the risk of bias and critical appraisal in the qualitative assessment of the articles are as follows. We have 8 experimental articles that meet the inclusion criteria for our study. These articles were selected based on their relevance to our research question and their adherence to our predetermined criteria for experimental design. We evaluated the articles based on the JBI critical appraisal checklist (Fig. 2). Question four (Were participants blind to treatment assignment?) And question five (Were those delivering treatment blind to treatment assignment?) Were not applicable for most of the studies as in the communicate based intervention it is not feasible for the participants and the person who deliver the services to be blind of interventions. The rest of the items were enough qualified to rely on the results (Fig. 2). The result of the quasi experimental and economy studies checklists depicted in the Supplementary Material 1:Appendix.

### The demographic characteristics of included articles

Demographic characteristics of included articles revealed Table 1.

**Table 1:** The characteristics of included articles

**Row**	**Articles**	**Design**	**City**	**Gender of samples**	**Number of samples**			**Follow-up (month)**	**Intervention**	**Type of intervention**	Tools*
					**Intervention group 1**	**Intervention group 2**	**Control**				
1.	Moradi (2017) [13]	RCT	Tehran	Women & Men	60	60	60	20	Telephone follow-up or home visit, in addition to caregivers’ education and training of social skills	Community-based intervention	PANSS, YMRS, HDRS, CGI, GAF, CSQ, WHOQOL BREF
2.	Barekatan (2013) [11]	RCT	Isfahan	Women & Men	61	-	62	12	Follow-up phone calls, home visits, and psychoeducation for families	Community-based intervention	YMRS, HDRS, PANSS, CGI, GAF, WHO-QOL
3.	Malakouti.1(2016) [12]	RCT	Tehran	Women & Men	60	61	61	12	Home visit & telephone follow up	Community-based intervention	KELS, KQC, FEIS, GHQ-28, CQS, PANSS, YMRS, burden
4.	Sharifi (2012) [14]	RCT	Tehran	Women & Men	66	-	64	12	Home visit	Community-based intervention	PANSS, YMRS, GAF, whoqol, CSQ
5.	Sadeghi Babokani (2020) [15]	Quasi-experimental	Isfahan	Women	15	15	15	0	Behavioral Activation & Psychosocial Rehabilitation	Psychological intervention	MOCA
6.	Malakouti.2 (2009) [16]	RCT	Tehran	Women & Men	117	-	-	12	Home visit case management two groups (family1 and health worker2)	Community-based intervention	GHQ, KELS, FEIS
7.	Mohebi (2017) [17]	RCT	Tehran	Men	20	-	20	3	Community re-entry program	Community-based intervention	MARS
8.	Akbari (2017) [18]	Quasi-experimental	Sanandaj	Men	15	-	15	0	Psychosocial Rehabilitation (12 weeks)	Psychological intervention	ANSQ—DSS
9.	Fayyazi Bordbar (2020) [19]	Quasi-experimental	Mashhad	Women & Men	29	-	28	12	A psycho-educational training session	Psychological intervention	The number of psychiatric visits, relapse status, number of re-hospitalizations, and time to relapse
10.	Ahmadi (2020) [20]	Quasi-experimental	Tehran	Men	15	-	15	6	Family Psychological Training	Psychological intervention	PANSS
11.	Malakouti.3 (2015) [21]	RCT	Tehran	Women & Men	46	57	57	12	The clients and their caregivers received monthly home visits (education and treatment supervision)	Community-based intervention	KELS, KQC, FEIS, GHQ-28, CQS, PANSS, YMRS
12.	Shahmiri (2014) [22]	Quasi-experimental	Tehran	Men	10	-	10	0	The sub-programs of social living skills and it is independent	Psychological intervention	MESS
13.	Mohammadzadeh (2012) [23]	RCT	Tehran	Men	12	-	12	0	Community Re-entry Program (CRM)	Community-based intervention	PANSS
14.	Fallahi-Khoshknab (2007) [24]	RCT	Tehran	Men	24	-	24	3	Home visit (for 3 month)	Community-based intervention	BPRS
15.	Jamshidi (2017) [25]	Quasi-experimental	Hamedan	Women & Men	10	-	10	0	Community re-entry program	Community-based intervention	ACIS, PANSS
16.	Dashtbozorgi (2009) [26]	RCT	Ahvaz	Women & Men	17	-	14	3	Psychoeducation sessions (6 weeks)	Psychological intervention	MMFQ, HRSD, BRMS, GAF
17.	Hojjati-Abed (2010) [27]	Quasi experimental	Tehran	Women & Men	24	-	50	0	Psychosocial occupational therapy interventions	Community-based intervention	WQOLCQ

^*^The Positive and Negative Symptoms Scale (PANSS) for psychotic symptoms, the Young Mania, Rating Scale (YMRS) for manic symptoms, the Hamilton Depression Rating Scale (HDRS) for depressive symptoms, the Clinical Global Impression (CGI) for illness severity and the Global Assessment of Functioning (GAF) Scale, Client Satisfaction Questionnaire (CSQ) tool, World Health Organization Quality of Life-BREF (WHOQOL BREF) Questionnaire, Knowledge Questionnaire for caregivers (KQC), Family Experience Interview Schedule (FEIS), Medication Adherence Rating Scale (MARS), Andreasen negative symptoms questionnaire (ANSQ), Dehbozorgi social skills(DSS), Kohlman Evaluation of Living Skills (KELS), General Health Questionaire-28(GHQ-28), Client Questionnaire Satisfaction (CQS), Matson evaluation of social skills (MESS), Brief Psychiatric Rating Scale (BPRS), Assessment of Communication and Interaction Skills (ACIS), Mc Master Family Questionnaire (MMFQ), Beach-Rafaelsen Mania Scale(BRMS), Wisconsin Quality of Life Client Questionnaire (WQOLCQ)

The results of intervention comparing before and after 12-months follow-up presented. The findings related to the tools used in intervention studies are presented in Table 2. As noted, most tools reported a weak level of heterogeneity(I^2^), so the meta-analysis has not been performed. The meta-analysis was conducted for rehospitalization, PANSS (Positive and Negative Syndrome Scale) as a psychopathology assessment tool and KELS (Kohlman Evaluation of Living Skills of the patients) and FEIS variables indicating psychological distress in caregivers.

**Table 2:** The effectiveness of intervention for the period of 12 months follow-up

**Tools**	**Follow-up**	**Standardized mean difference (SMD) (95% CI)**	***P*-value**	**I**^**2**^**%**	**No. of study in analysis**	**Comments**
**Tools used to evaluate the effectiveness of interventions for caregivers**						
CGI	12 months	-1.731 (-3.932, 0.469)	0.23	97.982	2	Comparing Mean diff of control and intervention during the follow up—home visit/telephone-follow up
CSQ	12 months	0.547 (-0.02, 1.114)	0.059	85.22	3	Comparing Mean diff of control and intervention during the follow up—home visit/telephone-follow up
GAF	12 months	0.221 (-0.604, 1.047)	0.599	93.99	3	Comparing Mean diff of control and intervention during the follow up—home visit/ telephone-follow up
KELS	3-month	-0.707 (-0.978, -0.436)	< 0.001	0.00	2	Comparing Mean diff of control and intervention during the follow up—home visit/telephone-follow up– nurse
PANSS	12 months	-0.310 (-0.489, -0.131)	0.001	40.22	5	Comparing Mean diff of control and intervention during the follow up—home visit/telephone-follow up– nurse & psychologist
WHO QOL	3-month	-0.246 (-0.500, 0.007)	0.057	71.13	2	Comparing Mean diff of control and intervention during the follow up—home visit/telephone-follow up
YMRS	3-month	-0.764 (-1.274, -0.253)	0.003	85.23	4	Comparing Mean diff of control and intervention during the follow up—home/telephone-follow up
GHQ	3-month	-0.570 (-1.235, 0.095)	0.093	83.50	2	Comparing Mean diff of control and intervention during the follow up—home visit/telephone-follow up
**Tools used to evaluate the effectiveness of interventions for patients**						
FEIS^a^	3-month	-0.554 (-0.990, -0.103)	0.016	63.22	2	Comparing Mean diff of control and intervention during the follow up—home visit/telephone-follow up
KQC	12 months	0.727 (-0.154, 1.608)	0.106	93.57	3	Comparing Mean diff of control and intervention during the follow up—home visit/telephone-follow up – nurse & psychologist

^a^*FEIS* Family Experience Interview Schedule, *KQC* Knowledge Questionnaire for caregivers, *CGI* Clinical Global Impression, *CSQ* Client Satisfaction Questionnaire, *GAF* Global Assessment of Functioning, *HDRS* Hamilton depression rating scale, *KELS* Kohlman Evaluation of Living Skills, *PANSS* Positive and Negative Syndrome Scale, *WHO QOL* Wisconsin Quality of Life Client Questionnaire, *YMRS* Young Mania Rating Scale

### The rehospitalization rate

**Fig. 3:**
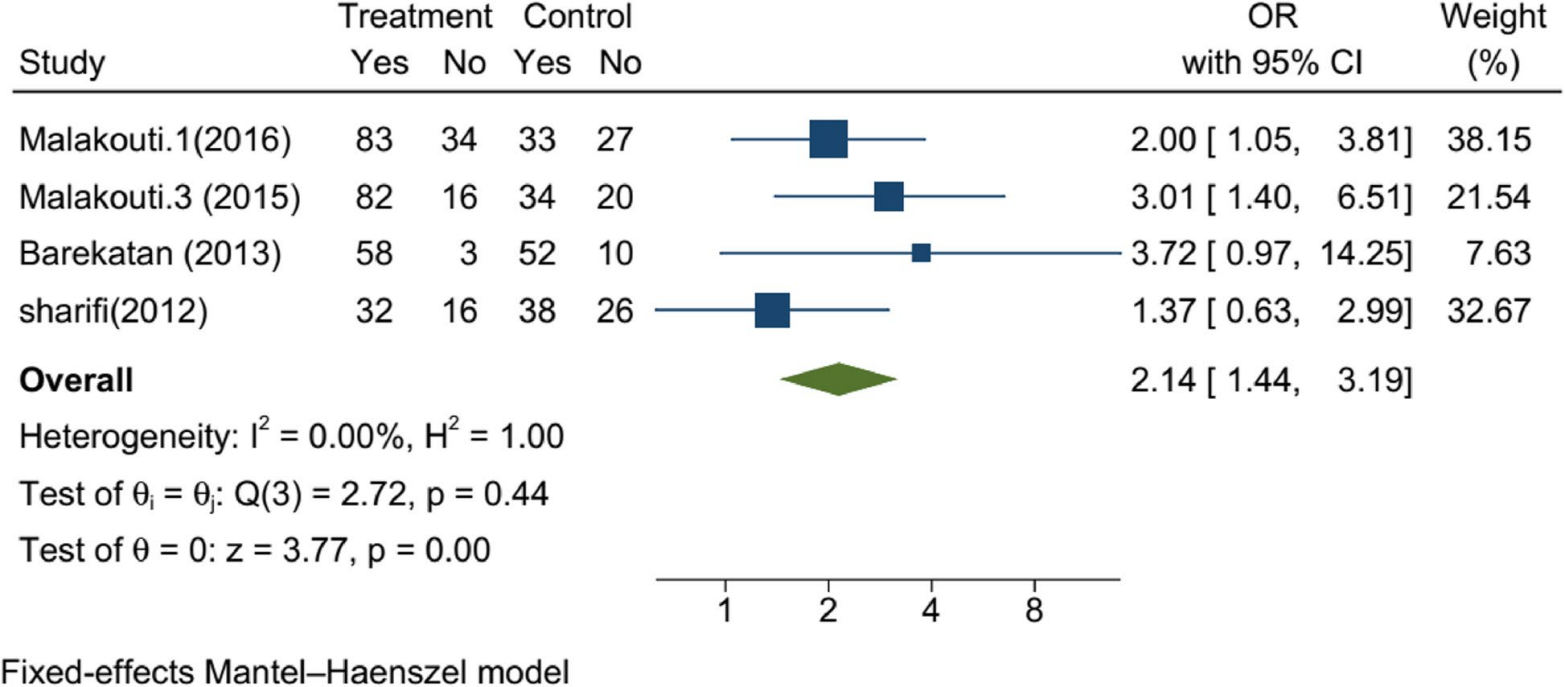
The result of the meta-analysis of rehospitalization among four studies

**Fig. 4:**
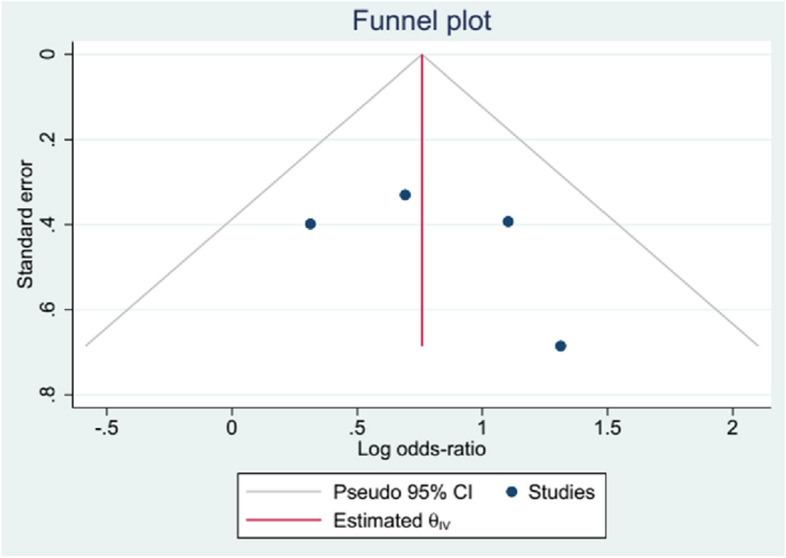
Funnel plot, publication bias for rehospitalization in studies

One of the study's primary goals was to evaluate the effect of any CBMHS on rehospitalization after the index discharge. The CBMHS included home-visit and telephone follow-ups, the rehospitalization rate among patients who received CBMHS (with a total of 595 participants in both intervention and control groups) was 2.14 times lower compared to those who received treatment as usual (OR: 2.14,95%CI: 1.44, 3.19). Mohebi [17] was the only article that used Medication Adherence Rating Scale (MARS) to evaluate the compliance of the patient with treatment (SMD: 3.15, CI: 95% 2.31, 3.98). The result of the meta-analysis of rehospitalization among four studies showed in Fig. 3. There was not any publication bias. It shown as Fig. 4.

### Severity of psychopathology

**Fig. 5:**
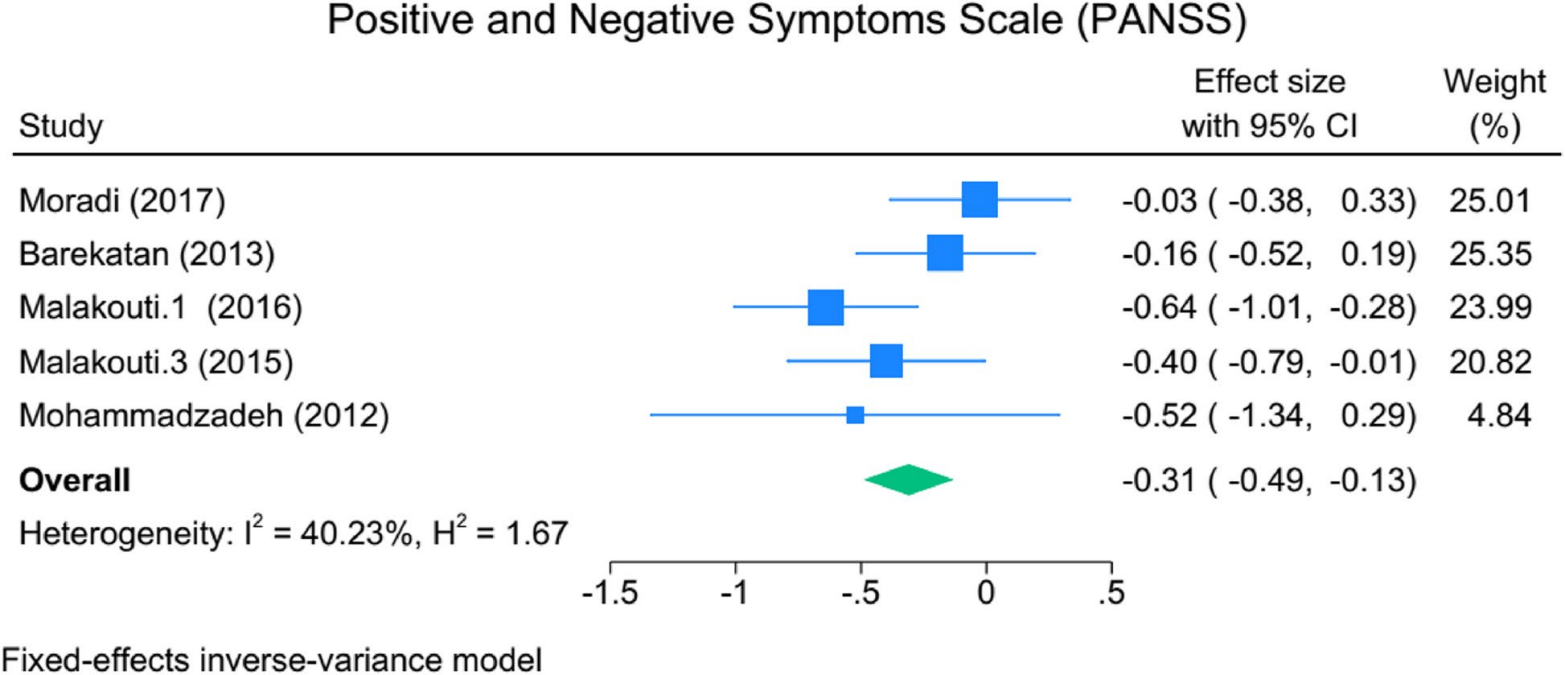
The meta-analysis of the studies for PANSS

Out of 17 studies, 5 used PANSS to evaluate the effect of intervention on psychopathology, whose data were amenable to analysis (with a total of 669 participants in both intervention and control groups). Meta-analysis shows that after 12 months of intervention, CBMHS are successful in reducing significantly of the severity of psychopathology (SMD: -0.31, 95%CI: -0.49 to -0.13, I^2^ = 40.23%). Akbari [18] was the only one that used ANSQ -Anderson Negative Symptoms- (SMD: -0.581, 95%CI: -1.312, 0.149). The meta-analysis of the studies for PANSS showed in Fig. 5.

The result of meta-analysis on the YOUNG (evaluating the severity of Bipolar mood disorder) shows significant difference by implementing the intervention (SMD: -0.764, 95% CI: -1.274, -0.253, I^2^ = 85.22%), however the heterogeneity among four studies were not acceptable.

Upon visual examination of the funnel plot, no significant signs of asymmetry were observed (located in the Supplementary Material 1: Appendix).

Social skills as secondary outcomes of the study could be considered as one of the outcomes of any intervention that aims to help the patient to be as independent as possible in the family and society. Just two studies used KELS to evaluate the social skills of the patients. The result of KELS shows a high effect size (SMD: -0.7, 95%CI: -0.98 to -0.44, I2 = 0.00%), and the community-based interventions are more promising. The same result was revealed by the study of Shahmiri (2014) by Matson evaluation of social skills (MESS) (SMD: -0.877, 95%CI: -1.749, 0.041) (lower scores indicating better functioning).

The tools of ACIS Assessment of Communication and Interaction Skills (mean difference 1.747 (CI: 1.08, 2.41).) Higher scores indicate better skills, such as DSK Dehbozorgi's social skills (SMD: 0.835, 95%CI: 0.088, 1.581). (Lower scores indicate lower social skills).

The burden of the caregivers was evaluated by FEIS (evaluating the burden of the caregivers) in the meta-analysis (not shown in the article). It shows that again in the two articles, the effect size was -0.55 (SMD: -0.55, 95%CI: -0.99, -0.1, I2 = 63.2) (in favor of community-based services.

For the CSQ, which evaluated the satisfaction of the clients from the services, there were not any significant differences with the control group. In the study of Sharifi [14], the quality of life of (WHOQOL) patients has been improved marginally (SMD:-0.246, 95%CI: -0.500, 0.007) (*P* = 0.057). However, in the study of Hojati- Abad [27] WQOLCQ (Wisconsin Quality of Life Client Questionnaire (SMD: 0.798, 95%CI: 0.29, 5, 1.301), there was not any significant difference.

### Economic evaluation studies

For economic evaluation we considered tow indexes reported incremental cost-effectiveness ratios (ICER) [28] and Quality-Adjusted Life Year (QUALY) [29, 30], which is are more common indexes economic evaluation.

The QALY serves as a metric for assessing the worth of health outcomes. As health is contingent upon both lifespan and well-being, the QALY was formulated as an endeavor to amalgamate the value of these attributes into a solitary index. In the field of mental health, improving the quality of life for patients and reducing the burden of the disease not only for the patients themselves but also for their families and society as a whole. QALYs can be integrated with medical expenses to derive a final universal measure of cost/QALY. This parameter facilitates the comparison of the cost-effectiveness of various treatments without bias.

In numerous healthcare systems, determinations regarding the reimbursement and availability of new medications hinge upon health technology assessments. These assessments, involve the evaluation of an ICER. Decision-makers then weigh the ICER against a predetermined benchmark for cost-effectiveness, referred to as the cost-effectiveness threshold (CET), in order to ascertain whether reimbursement should be granted or withheld [28].

We identified two reports that met our inclusion criteria concerning economic evaluations of community-based interventions to improve the mental health of individuals with SMI [13, 21]. In both studies were included QUALY and ICER.

Malakouti et al. ICER for aftercare home services following the discharge of individuals with SMI. Their analysis was based on a 12-month follow-up of participants in a clinical trial conducted between 2007 and 2008. They found that the ICER was 5.7 million Rials (IRR) per QALY when using general practitioners (GPs) as care providers during home visits and 5.0 million IRR per QALY when replacing GPs with nurses [13]. In a separate study, Moradi-Lakeh et al. conducted a cost-utility analysis of aftercare services following the discharge of individuals hospitalized for SMI. Their analysis was based on a clinical trial performed from 2012 to 2014, with a 20-month follow-up. They reported an ICER of US$8,399 (95% CI: 8,178–8,620) per QALY for the intervention [13]. It is worth noting that the services provided by the second study were more comprehensive. In these two studies showed that community-based interventions can be useful in terms of cost–benefit and cost-effectiveness.

While the ICER measures in these two studies differed considerably (partly due to a significant fluctuation in IRR-USD exchange rates $1 = 935 to 10,402 IRR, and other services provided by the second study included the expenses of general psychologists, supervising psychiatrists, as well as the costs of weekly co-ordination meetings of home visit teams, costs of classes for caregivers' education, training of social skills), both were found to be below the World Health Organization's recommended threshold for cost-effectiveness of health interventions [13, 21]. The second study aimed to provide the cheapest and most effective intervention and evaluated the feasibility of providing such CBMHS. However, from $ 613 to $8400, it is feasible to provide such services while considering the exchange and inflation rates.

## Discussion

Out of 127 English and Persian language articles which were conducted in IRAN, seventeen met the eligibility criteria for inclusion in the systematic review and meta-analysis. community-based services effectively reduced rehospitalizations by 2times. reduction in psychopathology with moderate effect sizes. economic cost-effectiveness, with ICER values falling below the recommendations set by the WHO s.

Community psychiatry was launched in the USA in 1970 [31], community psychiatry was launched in the USA in 1970 [31] Reducing hospitalization, enabling individuals to have an active social life in society [32], and providing holistic care are among the main goals of community psychiatry community psychiatry was launched in the USA in 1970 [31].

CBMHS, including home visits, telephone follow-ups, case management systems, intensive care systems, and other CBMHS developed in different societies, have emerged and expanded to address this new challenge [32]. Even in a crisis, CBMHS mobile crisis intervention can reduce the number of readmissions [33]. Providing and connecting discharged patients to community services as soon as possible is a critical issue to prevent readmission [34].

On the other hand, the evidence shows that the direct daily costs for community-based social psychiatric care were about half the costs of inpatient treatment over the entire period [34, 35]. However, reducing the number of readmissions depends on the intensity of community services, and it may yield different results in some societies [36].

Some reasons may be nominated for reduction of rehospitalization [37, 38]. However, compared with the developed societies, we need to examine this issue from a dual perspective. First, in addition to having social activities and supporting the patients to have an almost independent life, second, the shortage of psychiatric beds is an essential matter in our country's mental health services. According to the according recommended assesment, we should have forty more than 56 thousand psychiatric beds in Iran [39, 40] which falls behindrrrr. With the best estimation, we have thirty thousand psychiatric beds, of which fifty percent belong to the Welfare Organization for long-term hospitalization and rehabilitation [40]. Therefore, an extension of CBMHS psychiatry could help the patient and their family but also help the national mental health service to compensate for the shortage of facilities. Exacerbating the severity of psychopathology could increase the odd behaviors and tension in the family, leading to the patient's hospitalization [41, 42]. Reducing the severity of psychopathology, as revealed in the study results as the severity of PANSS and YMRS in the current study, could be helpful. Increasing adherence to medication due to frequent contact (in person or by telephone) encourages the patient to comply with the treatment, which is the main reason for reducing the severity of psychopathology.

Having a long-duration mental health stability state without exacerbation of the symptoms, in addition to psychoeducation and behavioral rehearsal, which is part of community-based intervention, could provide suitable opportunities for the patient to retrieve his/her behavioral skills or learn new skills, which are necessary for independent living [43, 44]. Social skills, evaluated by KELS and MESS, showed promising outcomes for CBMHS with remarkable effect size. However, gaining social skills requires intense and close supervision, which any community-based intervention may not obtain, as shown by the study of Jamshidi [25]; the results of this study are controversial ACIS.

The burden and mental health of the caregivers for chronic diseases such as schizophrenia, bipolar mood disorder, and dementia are essential in mental health services. Given that in developing countries [45, 46], taking care of patients is on the shoulders of the families, providing aftercare services and regular contact with the patient and the caregivers could help them overcome the long-lasting problems in their family. The reduction of burden and improvement of the mental health condition of the caregivers show that such services have a positive impact on them [44].

Satisfaction with the services and quality of life, which did not exhibit significant differences from the control group in our study, remains a topic of extensive debate. Satisfaction and quality of life depend on physical and mental health and suitable living conditions such as housing, jobs, and income, which are not fulfilled by community-based services and are beyond the scope of these services and require intersectoral collaboration [12, 21, 38].

The challenge of the studies performed in Iran as CBMHS was using multiple tools with similar goals to evaluate corresponding variables. This hinders the ability to compare and analyze the results effectively. For instance, the study employed four different tools to measure life skills. Also, despite the history of CBMHS in Iran, there is a lower publication rate for the results of these interventions and programs. The expectation of having more articles that evaluate this specific area emphasizes the need for attention to be given to this issue.

## Conclusion

The findings of community-based studies in Iran, although rare, showed that in line with international studies, this type of service is compatible and effective in Iran's economic, social, and cultural conditions. Even though there are challenges regarding patient satisfaction and organizational interdepartmental cooperation. These findings emphasize that community-based services not only in Iran but also in similar countries should be preferred over long-term inpatient services.

### Supplementary Information

**Supplementary Material 1.**

## Data Availability

This is an evidence synthesis study, all data is available from the primary research studies, or can be circulated from the corresponding author.
