# Supplementary material for: Effectiveness and cost-effectiveness of community-based mental health services for individuals with severe mental illness in Iran: a systematic review and meta-analysis
Source: BMC Psychiatry. 2024 Apr 4;24:256. doi: 10.1186/s12888-024-05666-7 (PMC10993444; doi:10.1186/s12888-024-05666-7)
Supplement: Supplementary file 1 — Supplementary Material 1. [file 12888_2024_5666_MOESM1_ESM.docx]

**Appendix**

**A: search strategies for evaluating articles**

String (TITLE-ABS-KEY (("Mental Disorder" OR "Psychiatric Illness" OR "Psychiatric Disease" OR "Mental Illness" OR "Psychiatric Disorder" OR "Psychiatric Diagnosis"))) AND (ALL (Iran OR "Islamic Republic of Iran" OR "iriran" OR "I R. Iran" OR

Persia*)) AND ((TITLE-ABS-KEY ("Community Mental Health Services" OR "Community Mental Health" OR "Community Treatment" OR "Community-based")) OR

( TITLE-ABS-KEY ("Home visit" OR "home care" OR "domestic health care" OR "domiciliary care" OR "home health" OR "home help" OR "home service")) OR ( TITLE-ABS-KEY ("Telerehabilitation" OR telerehabilitation OR "Tele-rehabilitation" OR "Tele rehabilitation" OR "Remote Rehabilitation" OR "e-rehabilitation") ) ) AND ( LIMIT-TO ( DOCTYPE, "ar") ) AND ( LIMIT-TO ( PUBSTAGE, "final") ) AND ( LIMIT-TO (AFFILCOUNTRY, "Iran") ) AND ( LIMIT-TO ( LANGUAGE, "English") OR LIMIT-TO ( LANGUAGE, "Persian") )

**B: Risk of bias assessment**

**JBI Critical Appraisal Checklist for randomized Controlled trials**

| 1. Was true randomization used for assignment of participants to treatment groups? |
| --- |
| 1. Was allocation to treatment groups concealed? |
| 1. Were treatment groups similar at the baseline? |
| 1. Were participants blind to treatment assignment? |
| 1. Were those delivering treatment blind to treatment assignment? |
| 1. Were outcomes assessors blind to treatment assignment? |
| 1. Were treatment groups treated identically other than the intervention of interest? |
| 1. Was follow up complete and if not, were differences between groups in terms of their follow up adequately described and analysed? |
| 1. Were participants analysed in the groups to which they were randomized? |
| 1. Were outcomes measured in the same way for treatment groups? |
| 1. Were outcomes measured in a reliable way? |
| 1. Was appropriate statistical analysis used? |
| 1. Was the trial design appropriate, and any deviations from the standard RCT design (individual randomization, parallel groups) accounted for in the conduct and analysis of the trial? |

**JBI Critical Appraisal Checklist for quasi-experimental studies**

| 1. Is it clear in the study what is the cause’ and what is the ‘effect’ (i.e., there is no confusion about which variable comes first)? |
| --- |
| 1. Were the participants included in any comparisons similar? |
| 1. Were the participants included in any comparisons receiving similar treatment/care, other than the exposure or intervention of interest? |
| 1. Was there a control group? |
| 1. Were there multiple measurements of the outcome both pre and post the intervention/exposure? |
| 1. Was follow up complete and if not, were differences between groups in terms of their follow up adequately described and analyzed? |
| 1. Were the outcomes of participants included in any comparisons measured in the same way? |
| 1. Were outcomes measured in a reliable way? |
| 1. Was appropriate statistical analysis used? |

**JBI Critical Appraisal Checklist for economic evaluations**

| 1. Is there a well-defined question? |
| --- |
| 1. Is there comprehensive description of alternatives? |
| 1. Are all important and relevant costs and outcomes for each alternative identified? |
| 1. Has clinical effectiveness been established? |
| 1. Are costs and outcomes measured accurately? |
| 1. Are costs and outcomes valued credibly? |
| 1. Are costs and outcomes adjusted for differential timing? |
| 1. Is there an incremental analysis of costs and consequences? |
| 1. Were sensitivity analyses conducted to investigate uncertainty in estimates of cost or consequences? |
| 1. Do study results include all issues of concern to users? |
| 1. Are the results generalizable to the setting of interest in the review? |

Fig.6: Quality assessment of quasi-experimental studies

Fig.7: Quality assessment of economic evaluation studies

**C: publication bias**

Fig.8: publication bias for PANSS


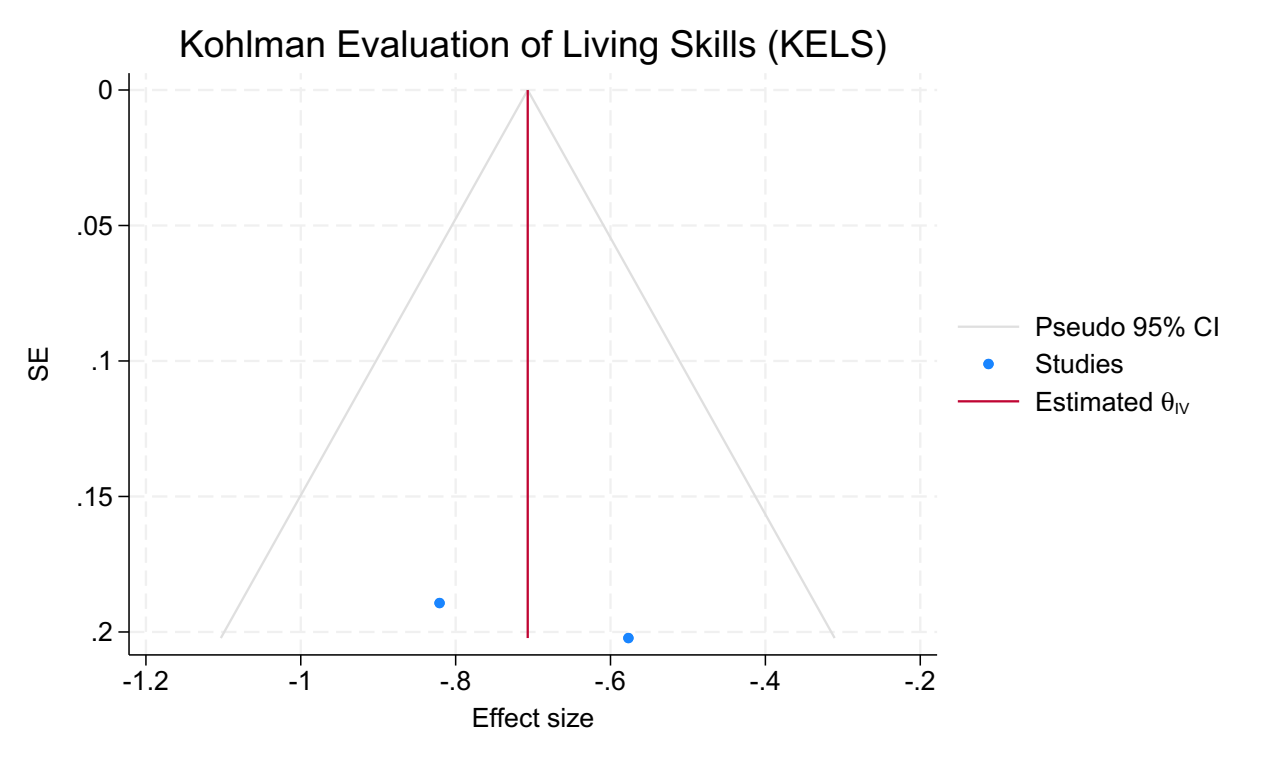


Figure 9: publication bias for KELS

**D: Other tools**

Table 3: The tools that were utilized solely in one article

| Tool | Standardized mean difference (95 % CI) |
| --- | --- |
| ACIS | 1.747 (1.08, 2.41 ) |
| ANSQ | -0.581 (-1.312, 0.149) |
| BRPS |  |
| DSK | 0.835 (0.088, 1.581) |
| MARS | 3.146 (2.310, 3.983) |
| WQOLCQ | 0.798 (0.295, 1.301) |
| MESS | -0.877 (-1.749, 0.041) |

Assessment of Communication and Interaction Skills (ACIS), Anderson Negative Symptoms (ANSQ), Brief Psychiatric Rating Scale (BPRS), Dehbozorgi social skills (DSK), general health of caregivers (GHC), Medication Adherence Rating Scale (MARS), Wisconsin Quality of Life Client Questionnaire (WQOLCQ), Matson evaluation of social skills (MESS

**E: A brief description of some of the tools used**

1. Experience Interview Schedule (FEIS)

An instrument for measuring the burden of caregivers. A higher score indicates a higher burden of illness, and a lower score indicates a lower burden of illness.

1. The Clinical Global Impressions Scale (CGI)

A measure used by healthcare professionals to assess the severity of mental illness and the effectiveness of treatment. It is a simple tool that involves rating the patient's overall level of functioning on a scale from 1 to 7, with lower scores indicating better functioning (9).

1. The Client Satisfaction Questionnaire (CSQ)

A tool used to assess the satisfaction of clients with mental health services. It consists of a series of questions that measure different aspects of the client's experience, such as the quality of care, the therapist-client relationship, and the outcome of treatment. Higher scores indicate greater satisfaction with mental health services. The CSQ is typically scored on a scale from 1 to 4 or 1 to 5, with higher scores indicating higher levels of satisfaction. However, the specific scoring system can vary depending on the version of the CSQ being used. In general, a score of 4 or 5 on the CSQ suggests that the client is very satisfied with their mental health services, while a score of 2 or lower indicates dissatisfaction (10).

1. Hamilton depression rating scale (HDRS)

A widely used tool to measure the severity of depressive symptoms in individuals with depression. Higher scores indicating greater severity of symptoms.

1. Knowledge Questionnaire for caregivers (KQC)

A questionnaire to assess caregiver mental health, burden and knowledge about mental illness and its treatment. It consists of a series of questions designed to measure the caregiver's understanding of various aspects of mental illness, including symptoms, causes, treatments, and medications (11).

Kohlman Evaluation of Living Skills (KELS)

1. A tool used to evaluate an individual's ability to perform basic living skills. A higher score indicates better function and greater independence in performing activities of daily living (adls) and instrumental activities of daily living (iadls).
2. Positive and Negative Syndrome Scale (PANSS)

A questionnaire to evaluating positive and negative symptoms subscale respectively. Higher score reflected more severe symptoms.

1. Wisconsin Quality of Life Client Questionnaire (WHO QOL)

A widely used assessment tool designed to measure an individual's perceived quality of life. Is scored on a Likert-type scale, typically ranging from 1 to 5 or 1 to 7, with higher scores indicating better quality of life.

1. Young Mania Rating Scale (YMRS)

A tool used to measure the severity of manic symptoms in individuals with bipolar disorder. Higher scores indicating more severe manic symptoms.
